# A Comparitive Assessement of Cytokine Expression in Human-Derived Cell Lines Exposed to Alpha Particles and X-Rays

**DOI:** 10.1100/2012/609295

**Published:** 2012-04-22

**Authors:** Vinita Chauhan, Matthew Howland, Ruth Wilkins

**Affiliations:** Consumer and Clinical Radiation Protection Bureau, Health Canada, Ottawa, ON, Canada K1A 0K9

## Abstract

Alpha- (**α**-) particle radiation exposure has been linked to the development of lung cancer and has been identified as a radiation type likely to be employed in radiological dispersal devices. Currently, there exists a knowledge gap concerning cytokine modulations associated with exposure to **α**-particles. Bio-plex technology was employed to investigate changes in proinflammatory cytokines in two human-derived cell lines. Cells were irradiated at a dose of 1.5 Gy to either **α**-particles or X-rays at equivalent dose rates. The two cell lines exhibited a unique pattern of cytokine expression and the response varied with radiation type. Of the 27 cytokines assessed, only vascular endothelin growth factor (VEGF) was observed to be modulated in both cell lines solely after **α**-particle exposure, and the expression of VEGF was shown to be dose responsive. These results suggest that certain proinflammatory cytokines may be involved in the biological effects related to **α**- particle exposure and the responses are cell type and radiation type specific.

## 1. Introduction

Alpha- (*α*-) particle radiation has become an increasing public health concern, primarily due to emerging epidemiological evidence showing adverse health effects in relation to exposure to radon (^222^Rn) gas, an *α*-particle emitter (reviewed in [1]). It has been shown that ^222^Rn gas constitutes about half of the natural ionizing radiation dosage to which the average person is exposed [2] and has been epidemiologically linked as being the second leading cause of lung cancer after smoking [3]. In addition to being an environmental concern, *α*-particle emitting isotopes (i.e., ^241^Americium, ^210^Polonium, and ^238^Plutonium) are the most likely radionuclides to be employed in a terrorist precipitated mass-casualty event involving radiological dispersal devices (RDDs) [4]. A case in 2006 involved the poisoning of a specific individual using tea containing radioactive ^210^Po [5]. This has focused attention on the threats posed by radiological terrorism and the implications of such terrorist threats for radiation-accident preparedness and long-term biological consequences. As a result, there has been heightened interest in developing field-deployable bioassays for triage assessment and to better quantify the biological damage caused by such exposures. Clinically, *α*-particles are also emerging in treatment modalities for cancer therapy and in nuclear medicine [6, 7]. Though these modalities appear promising, the long-term biological risks associated with such exposures are unknown.

Research into biological effects related to *α*-particle radiation have been underway for the past decade, and the majority of these studies have focused on assessing cytogenetic endpoints (reviewed in [8]). However, there are limited studies that have assessed overall effects of *α*-particle radiation on protein modulations. In recent years, new approaches involving multiplexing technologies have evolved to allow the simultaneous analysis of the secretome [9]. Through the use of these techniques, biological effects associated with an insult can be studied within a microenvironment. This is important, as at least 20% of cancers stem from chronic inflammation, and even those that do not have altered microenvironment cytokine profiles (reviewed in [10]).

A decade ago, Narayanan et al. [11] examined interleukin- (IL-) 8 secretions in *α*-particle-irradiated normal human lung fibroblasts. This study was the first to show modulations in a chemoattractant cytokine following exposure to *α*-particles. The authors concluded that IL-8 induced by *α*-particles may contribute to an inflammatory response in the lower respiratory tract. Since then, only one other study has examined the cellular response to *α*-particle radiation at the intracellular proteomic level [12]. This study showed modulations in a limited number of proteins at high exposure levels (100–400 working level months (WLM)) in rats, and no analysis was conducted on the secretome. The advantages of assessing effects on secretory proteins is that it provides insight into mechanisms of *α*-particle-induced deleterious effects and identifies potential biomarkers of exposure. Therefore, the focus of this study was to assess biological changes in the protein microenvironment following *α*-particle radiation and in the process potentially identify biomarkers of such an exposure. To achieve these goals, secreted proteins were analyzed using Bio-plex antibody technology in two cell lines representative of epithelial lung tissue and surrounding monocytic cells.

## 2. Materials and Methods

### 2.1. Cell Culture and Exposures

A human-derived lung epithelial cell line (A549) with a doubling time of ~22 hours and a human-derived peripheral blood monocytic cell line (THP-1) with a doubling time of ~26 hours were obtained from American Type Culture Collection (ATCC, Manassas, VA, USA). Cells were maintained in a humidified incubator (37°C, 5% CO2/95% air) in 75 cm^2^ tissue culture flasks (Costar, Cambridge, MA, USA). THP-1 cells were grown in Royal Park Medical Institue-1640 (RPMI-1640) (Invitrogen Canada, Burlington, ON, Canada) in media containing 10% fetal bovine serum (FBS) (Sigma-Aldrich Canada, Oakville, ON, Canada). A549 cells were cultivated in F-12K medium (Invitrogen), containing 10% (FBS) (Sigma-Aldrich Canada). For the *α*-particle exposures, cells were cultured in thin Mylar-based plastic dishes (MD) (Chemplex Industries, Palm City, FL, USA), which allowed the penetration of the *α*-particles as previously described [13]. Cell viability was assessed from a 30 *μ*L aliquot both prior to exposure and immediately after exposure at all doses by the Trypan Blue viability assay (Invitrogen). A total of 1.0 × 10^6^ cells were seeded into 2 mL of culture media containing 100 units/mL of penicillin and 100 *μ*g/mL of streptomycin (Invitrogen Canada Inc.). The cells were cultured to ~90% confluency then exposed to *α*-particle radiation at doses ranging from 0.0 (control) to 1.5 Gy, using ^241^Am electroplated discs (Eckert and Ziegler Isotope Products Ltd, Valencia, CA, USA) having an activity level of 66.0 kBq ±3% (dose rate of 0.98 ± 0.01 Gy/h, linear energy transfer (LET) of 127.4 ± 0.4 keV/*μ*m). The absorbed dose of *α*-radiation to which cells were exposed was calculated using the GEANT4 v.9.1 Monte Carlo toolkit [13]. Cells destined for X-radiation at doses of 0 Gy and 1.5 Gy were exposed using the X-RAD 320 X-ray irradiation system at a dose rate of 0.98 ± 0.05 Gy/h, 120 keV (Precision X-ray, Inc., North Branford, CT, USA).

### 2.2. Bio-Plex Assay

Twenty-four hours following exposures, supernatants (containing 1X Halt Protease Inhibitor) (Thermo Fisher Scientific, MA, USA) from exposed (1.5 Gy *α*-particle and X-rays) and control samples were analyzed for secretion levels of 27 cytokines including interleukin- (IL-) 1b, IL-1ra, IL-2, IL-4, IL-5, IL-6, IL-7, IL-8, IL-9, IL-10, IL-12, IL-13, IL-15, IL-17, eotaxin, fibroblast growth factor (FGF), granulocyte-colony-stimulating factor (G-CSF), granulocyte/macrophage colony-stimulating factor (GM-CSF), interferon-*γ* (IFN-*γ*), interferon gamma-induced protein 10 kDa (IP-10), monocyte chemotactic protein-1 (MCP-1), macrophage inflammatory protein- (MIP-) 1a, MIP-1b, platelet-derived growth factor- (PDGF-) bb, regulated upon activation, normal *t*-cell expressed, and secreted (RANTES), tumour necrosis factor-*α* (TNF-*α*), and vascular endothelial growth factor (VEGF), using a multiplex assay as prepared according to the manufacturer's instructions (Bio-Rad). Briefly, conjugated beads were allowed to react with a sample containing a known (standard) or unknown amount of cytokines for thirty minutes. Conjugated beads with bound target were then washed and incubated with biotinylated detection antibodies that were directed against specific cytokine epitopes. The resulting complexes were then incubated for a further 10 min with streptavidin-phycoerythrin, and excess reagent was washed off and assessed for bound cytokine using a microtiter plate reader (Bio-Rad). The concentration of cytokines in supernatants was then assessed from the generated standard curves for each individual cytokine using Bio-Plex software (Bio-Rad).

### 2.3. Vascular Endothelial Growth Factor (VEGF) Enzyme Linked Immunosorbent Assay (ELISA)

Twenty-four hours following exposures, supernatants from exposed (0.5, 1.0, and 1.5 Gy) and control samples were analyzed for secretion levels of VEGF using commercially available ELISA set (Invitrogen). ELISA was performed according to the manufacturer's instructions. All samples and standards were measured in duplicate.

### 2.4. Statistical Analysis

Statistical differences (*P* ≤ 0.05) were inferred through use of a Student's *t*-test or one-way ANOVA with Dunnet's correction employing GraphPad InStat version 3.00 for Windows 95 (San Diego California, CA, USA, http://www.graphpad.com) for Bio-plex and ELISA samples, respectively. Analysis was based on an *n* = 5 independent biological experiments for Bio-plex and a separate *n* = 4 for ELISA samples.

## 3. Results

### 3.1. Secretome Analysis

Secreted proteins from cell cultures exposed to radiation were assessed using multiplex bead array technology (Table 1). Twenty-four hours after exposure, antibodies to a focused panel of 27 human inflammatory cytokines were incubated with media from cells exposed to *α*-particle radiation and X-rays. Statistical significance was ascribed to the results through use of a Student's *t*-test and no thresholds on fold changes. All results that were *P* < 0.05 were considered significant.

#### 3.1.1. A549

**Figure 1:**
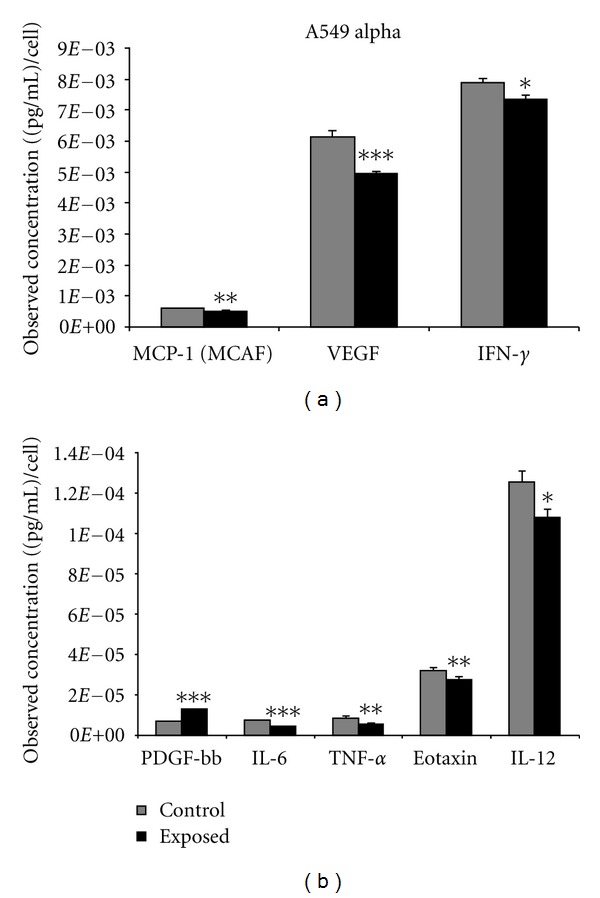
A549 cells were seeded into Mylar dishes and exposed to *α*-particle radiation at 0 and 1.5 Gy. A Bio-plex cytokine assay was performed quantitating 27 different cytokines, and the results showing statistical significance between control and exposed groups are presented. *represents *P* < 0.05, **represents *P* < 0.01, and ***represents *P* < 0.001, *n* = 5 biological replicates performed in duplicate. Bars are plotted as cell count corrected means ± SEM.

**Figure 2:**
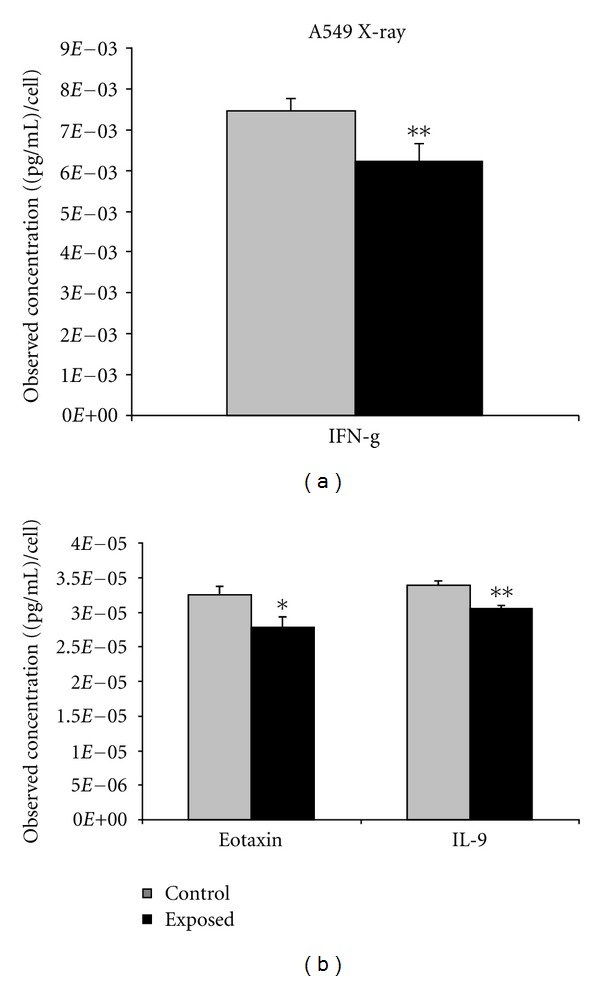
A549 cells were seeded into Mylar dishes and exposed to X-radiation at 0 and 1.5 Gy. A Bio-plex cytokine assay was performed quantitating 27 different cytokines, and the results showing statistical significance between control and exposed groups are presented. *represents *P* < 0.05, **represents *P* < 0.01, and ***represents *P* < 0.001, *n* = 5 biological replicates performed in duplicate. Bars are plotted as cell count corrected means ± SEM.

Of the 27 cytokines tested, all were detectable in the media, and 8 exhibited statistically significant differences between control and exposed treatment groups (Figure 1). Of these 8 cytokines, seven (IL-6, TNF-*α*, Eotaxin, IL-12, MCP-1, VEGF, and IFN-*γ*) were downregulated, and only one (PDGF-bb) was upregulated by ~2 fold. VEGF was observed to have relatively strong expression levels of ~6 × 10^−3^ (pg/mL)/cell in the control cells and was significantly downregulated (*P* < 0.001) in exposed cells. A comparison of the response to 1.5 Gy of X-irradiated cells showed a statistically significant downregulation in the levels of 3 cytokines (Eotaxin, IL-9, and INF-*γ*) (Figure 2). Of these three cytokines both Eotaxin and IFN-*γ* were shown to be common to both X-ray and *α*-particle-irradiated samples.

#### 3.1.2. THP-1

**Figure 3:**
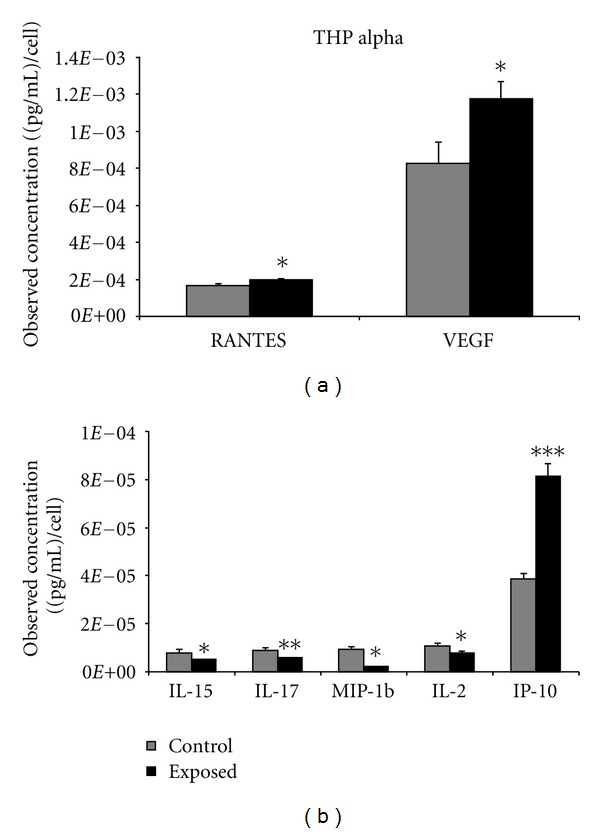
THP-1 cells were seeded into Mylar dishes and exposed to *α*-particle radiation at 0 and 1.5 Gy. A Bio-plex cytokine assay was performed quantitating 27 different cytokines, and the results showing statistical significance between control and exposed groups are presented. *represents *P* < 0.05, **represents *P* < 0.01, and ***represents *P* < 0.001, *n* = 5 biological replicates performed in duplicate. Bars are plotted as cell count corrected means ± SEM.

**Figure 4:**
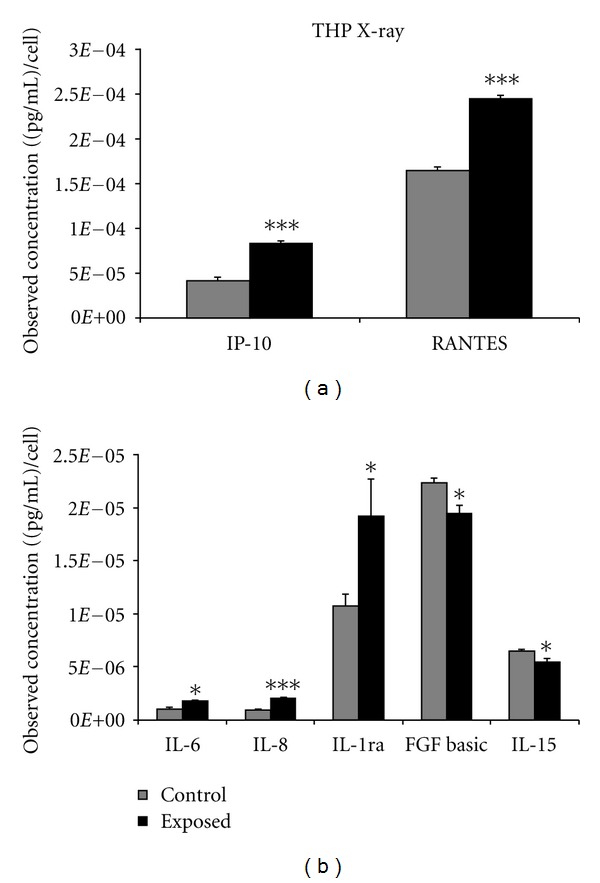
THP-1 cells were seeded into Mylar dishes and exposed to X-radiation at 0 and 1.5 Gy. A Bio-plex cytokine assay was performed quantitating 27 different cytokines, and the results showing statistical significance between control and exposed groups are presented. *represents *P* < 0.05, **represents *P* < 0.01, and ***represents *P* < 0.001, *n* = 5 biological replicates performed in duplicate. Bars are plotted as cell count corrected means ± SEM.

Analysis of a human blood monocytic cell line, THP-1, showed detectable amounts of all 27 cytokines. However, only 7 were found to be statistically significant and differentially expressed relative to the control group (*P* < 0.05). Among these 7 cytokines, 4 cytokines were downregulated (IL-15, IL-17, MIP-1b, and IL-2) and 3 cytokines were upregulated (IP-10, RANTES, and VEGF) following *α*-particle exposure (Figure 3). Similar to A549 cells, expression levels of VEGF were comparatively higher relative to the other cytokines which were shown to be expressed following *α*-particle treatment. The exposure of the THP-1 cells to X-rays elicited the secretion of 5 (IL-6, IL-8, IL-1ra, IP-10, and RANTES) upregulated cytokines and 2 (FGF basic and IL-15) downregulated cytokines. Of these, IL-15, IP-10, and RANTES were common to both radiation types and displayed a similar level of expression (Figure 4).

### 3.2. Heat Map of Cell-Line-Specific Responses

**Figure 5:**
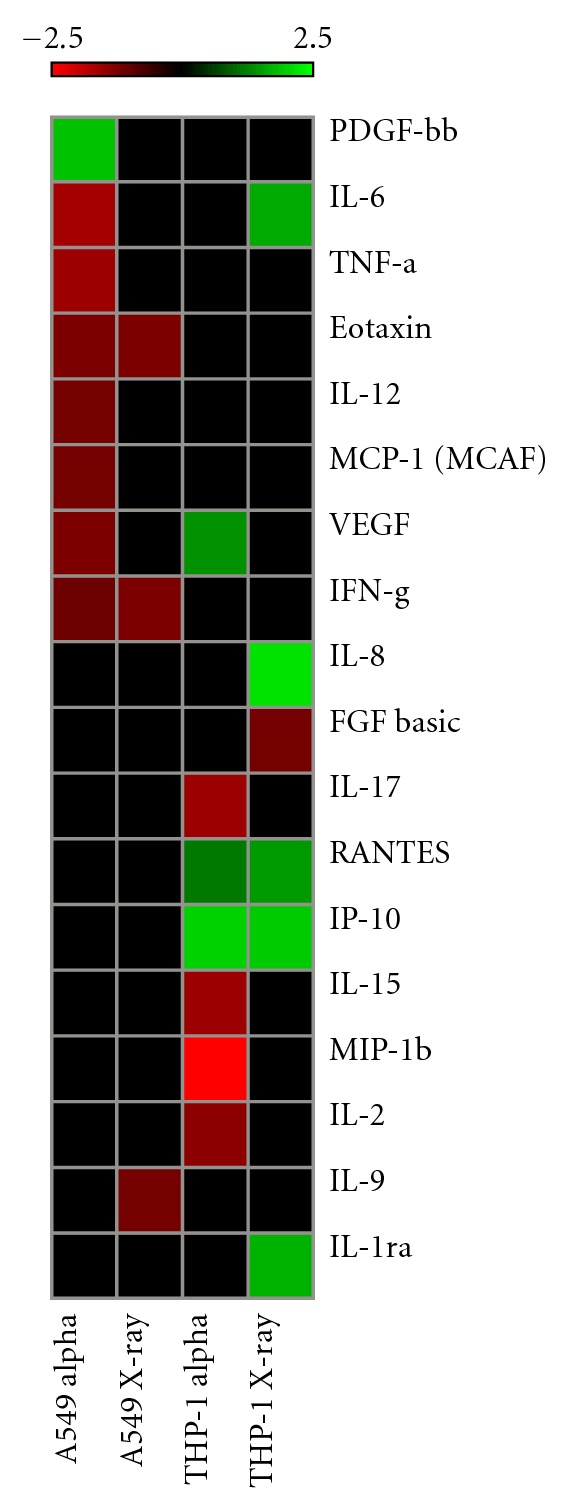
Heat map displaying cytokine fold changes for all cell lines assessed. Upregulation is represented with green shading and downregulation with red. Expression varies from −2.5 to 2.5.

A heat map was constructed to provide a qualitative representation of the similarities and differences in expression patterns of the cytokine responses obtained for the two different cell types and radiation types (Figure 5). Overall, each cell line displayed a unique *α*-particle-induced cytokine profile and minimal similarity in proinflammatory protein response was observed between the two radiation-types. Only VEGF was commonly modulated between the two cell types. However, VEGF expression was shown to be upregulated in THP-1 cells and downregulated in the A459 cells. VEGF was not differentially expressed in X-irradiated cells in either cell types.

### 3.3. ELISA Validation

**Figure 6:**
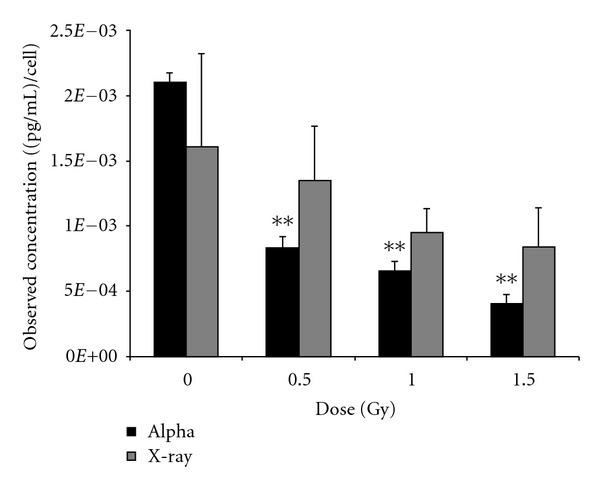
VEGF validation: twenty-four hours after exposure, cell culture supernatants were harvested from A549 cells exposed to 0, 0.5, 1.0, and 1.5 Gy of *α*-particle radiation and X-irradiation. 25 uL of sample was used to determine levels of VEGF secretion using ELISA. *represents *P* < 0.05, **represents *P* < 0.01, and ***represents *P* < 0.001, *n* = 5 biological replicates performed in duplicate. Bars are plotted as cell count corrected means ± SEM.

**Figure 7:**
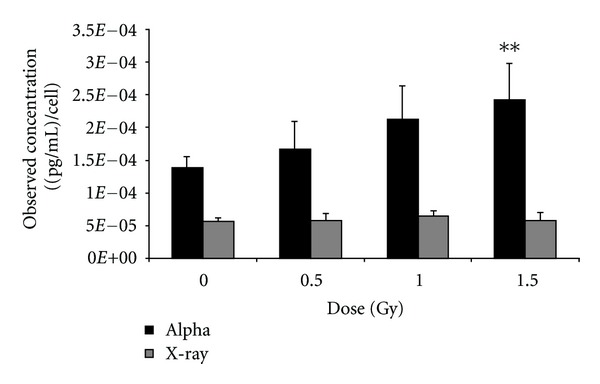
VEGF validation: twenty-four hours after exposure, cell culture supernatants were harvested from THP-1 cells exposed to 0, 0.5, 1.0, and 1.5 Gy of *α*-particle radiation and X-irradiation. 25 uL of sample was used to determine levels of VEGF secretion using ELISA. *represents *P* < 0.05, **represents *P* < 0.01, and ***represents *P* < 0.001, *n* = 5 biological replicates performed in duplicate. Bars are plotted as cell count corrected means ± SEM.

Among the cytokines that were screened for expression, only VEGF was observed to be commonly expressed in the two cell types following *α*-particle radiation. Thus, its response was further assessed at lower doses (Figure 6). A549 cells exposed to X-rays display a biological trend but not a statistically significant effect on VEGF expression using ELISA analysis. In contrast, A459 cells exposed to *α*-particle radiation showed a statistically significant decrease in VEGF expression. An analysis of VEGF response in THP-1 cells (Figure 7) exposed to *α*-particle radiation also showed VEGF expression to be dose responsive and specific to *α*-particle irradiated cells. For both cell types, the cytokine secretion responses were similar to that obtained using Bio-plex technology.

## 4. Discussion

The focus of this study was to delineate the biological effects of *α*-particle radiation at the secretome level. As the epithelial lung lining are the outermost cells exposed to inhaled environmental toxins and are the primary site of *α*-particle exposure, we focused our efforts on assessing protein modulations in this cell type. Further secretory screening was performed in a human monocytic circulating blood cell line which may be more amenable to biomarker discovery. In addition, the *α*-particle responses were contrasted with X-rays at an equivalent dose rate to better understand the biological impact of the two radiation types on inflammatory cytokine secretion. To date, there are a limited number of studies have examined cytokine secretion in *α*-particle exposed cells, and no studies that have conducted a comparative analysis of cytokine secretion following *α*-particles and X-irradiation at a similar dose rate. The results of this study show that A549 and THP-1 cells respond by the activation of different proinflammatory cytokines and this response is radiation type dependant. Overall, there was a low level of commonality observed in cytokine secretion, varying in terms of nature and expression level for each cell and radiation type.

Cytokines play an important role in the inflammatory response, as they are considered molecular messengers that have the potential to initiate tumour formation and progression (reviewed in [14]). Epithelial cells exposed to *α*-particles expressed cytokines related to both acute and chronic inflammation, predominately in the form of growth factors and interleukins. The majority of these cytokines were downregulated with the exception of PDGF-bb which was upregulated by ~1.5 fold in *α*-particle-irradiated cells. Upregulation in the expression of PDGF has been linked to neoplastic transformation and cancer metastasis [15]. Dysregulation of this cytokine in conjunction with VEGF, as was seen in this study, has also been linked to different types of malignancies (reviewed in [16]). In addition to the observed modulation in the expression of growth factors, the interleukins (IL-12, IL-6) were shown to be significantly downregulated along with MCP-1 and TNF-*α*. These cytokines have been shown to be involved in acute and chronic inflammation (reviewed in [17]). Although neoplastic transformation was not assessed in this study, chronic dysregulation of these cytokines has implications for cancer development (reviewed in [10]). 

A549 cells exposed to X-rays displayed a limited number of responding cytokines, with IL-9, a cytokine involved in chronic inflammation being uniquely expressed in this radiation type (reviewed in [18]). Only two cytokines were shown to be common amongst the radiation types. These included eotaxin and IFN-*γ*, both of which were downregulated following irradiation and have biological functions related to immunosurveillance and immunoregulation (reviewed in [19] and [20]). The limited number of cytokines expressed after X-ray exposure would imply a varying level of inflammation in comparison to *α*-particles. Studies have shown that DNA damage resulting from exposure to *α*-particle radiation is potentially more difficult to repair than low-linear energy transfer (LET) radiation (X-rays) and is more susceptible to mutagenic changes [21]. Primary human fibroblasts X-irradiated have shown the majority of the double strand breaks (DSBs) to be removed 24 after irradiation. In contrast, approximately 85% of the DSBs remain 24 h after *α*-particle exposure. Therefore, the cascade of events leading to the induction of proinflammatory responses may differ depending on the nature of insult as was observed in this study. The general downward trend in cytokine secretion following radiation exposure suggests a compromise of the A549 cells innate immunocapacity following irradiation, potentially priming cells to undergo apoptosis. A previous study from our laboratory has shown that monocytic cells to be 30% apoptotic, 96 h after *α*-particles exposure, only 5% of the cells are apoptotic after X-ray exposure [22].

To further assess if similar cytokine responses were obtained for an alternative cell type, the effects of radiation exposure were determined in a circulating cell type, THP-1, which maybe more suitable for biomarker discovery. As was observed in the A549 cells, significantly modulated cytokines in this cell line varied with radiation type. However, unlike the A549 cells, the human monocytic cells displayed a greater number of upregulated cytokines, potentially because of the innate properties of monocytic cells as an inflammatory cell-type. In THP-1 cells, three of the seven statistically significant cytokines modulated after *α*-particle exposure (RANTES, IL-15, and IP-10) were commonly expressed between the two radiation types. The remainder of the responding cytokines were unique to *α*-particle exposed cells and included IL-17, IL-2 MIP-1B, and VEGF.

There was no commonality in *α*-particle-induced cytokine secretions between A549 and THP-1 with the exception of VEGF. VEGF was modulated in both A549 and THP-1cells; however, its response was shown to be bidirectional, and this was validated at lower doses of radiation. It was observed that in THP-1 cells, VEGF expression was significantly upregulated, while in A549 cells, expression of this growth factor was downregulated. These modulations were observed to be dose responsive and specifically induced by *α*-particle insult, whereas X-irradiated cells showed no statistically significant changes in expression of VEGF in either cell type. VEGF's biological role as a vascular growth factor has been shown to be somewhat dichotomous, as it is needed for novel tissue vascularization but may promote tumour survival when dysregulated pathologically (reviewed in [23]). It is interesting to note that a VEGF response was invoked in two cell lines exclusively by *α*-particle insult and not X-rays. This suggests that VEGF may have an *α*-particle specific role though its bidirectional expression patterns may deter its use as a potential biomarker of exposure.

To summarize, *α*-particle radiation was shown to elicit specific cell-type secretory responses. A comparison of the responses with X-ray showed a limited number of comparable cytokines. Responding proinflammatory cytokines proteins were broadly related to interleukin family of proteins and growth factors. A number of the secreted proteins which were observed to be expressed following *α*-particle exposure have been shown to have clear links to neoplastic transformation and tumour promotion. Although in this study, no unique biomarkers of *α*-particle exposure were definitively identified, VEGF emerged as a candidate for further studies, as it was modulated exclusively by *α*-particle insult.

## Figures and Tables

**Table 1a:** (a)

Cell type		Cytokines screened													
		IL-1b	IL-1ra	IL-2	IL-4	IL-5	IL-6	IL-7	IL-8	IL-9	IL-10	IL-12	IL-13	IL-15	IL-17
A549-alpha (0 Gy)	Conc.	4.0*E* − 07	3.2*E* − 06	7.5*E* − 06	5.6*E* − 07	7.2*E* − 07	**7.5*E ***−** 06**	7.1*E* − 06	2.9*E* − 05	3.5*E* − 05	5.9*E* − 06	**1.3*E ***−** 04**	1.7*E* − 05	8.9*E* − 06	7.0*E* − 06
	SD	2.3*E* − 08	1.7*E* − 06	2.1*E* − 06	3.2*E* − 07	9.0*E* − 08	**6.0*E ***−** 07**	8.4*E* − 07	7.1*E* − 06	3.8*E* − 06	9.3*E* − 07	**1.2*E ***−** 05**	1.2*E* − 06	1.2*E* − 06	1.2*E* − 06
	SEM	1.0*E* − 08	7.5*E* − 07	9.3*E* − 07	1.4*E* − 07	4.0*E* − 08	**2.7*E ***−** 07**	3.7*E* − 07	3.2*E* − 06	1.7*E* − 06	4.2*E* − 07	**5.6*E ***−** 06**	5.5*E* − 07	5.4*E* − 07	5.2*E* − 07
A549-alpha (1.5 Gy)	Conc.	3.8*E* − 07	3.0*E* − 06	6.7*E* − 06	5.5*E* − 07	6.5*E* − 07	**4.6*E ***−** 06**	6.6*E* − 06	2.2*E* − 05	3.0*E* − 05	5.0*E* − 06	**1.1*E ***−** 04**	1.7*E* − 05	7.7*E* − 06	6.1*E* − 06
	SD	3.1*E* − 08	2.3*E* − 06	2.3*E* − 06	2.8*E* − 07	3.2*E* − 08	**3.8*E ***−** 07**	1.0*E* − 06	3.8*E* − 06	2.9*E* − 06	1.1*E* − 06	**9.1*E ***−** 06**	1.4*E* − 06	7.2*E* − 07	4.9*E* − 07
	SEM	1.4*E* − 08	1.1*E* − 06	1.0*E* − 06	1.3*E* − 07	1.4*E* − 08	**1.7*E ***−** 07**	4.7*E* − 07	1.7*E* − 06	1.3*E* − 06	4.8*E* − 07	**4.1*E ***−** 06**	6.1*E* − 07	3.2*E* − 07	2.2*E* − 07
A549-X-ray (0 Gy)	Conc.	4.2*E* − 07	3.2*E* − 06	7.9*E* − 06	5.4*E* − 07	6.8*E* − 07	6.7*E* − 06	6.3*E* − 06	3.0*E* − 05	**3.4*E ***−** 05**	5.8*E* − 06	1.3*E* − 04	1.8*E* − 05	9.0*E* − 06	6.6*E* − 06
	SD	8.2*E* − 08	1.5*E* − 06	1.9*E* − 06	2.9*E* − 07	9.0*E* − 08	4.3*E* − 07	1.2*E* − 06	8.2*E* − 06	**1.4*E ***−** 06**	9.5*E* − 07	1.2*E* − 05	2.8*E* − 06	1.4*E* − 06	7.1*E* − 07
	SEM	3.7*E* − 08	6.8*E* − 07	8.5*E* − 07	1.3*E* − 07	4.0*E* − 08	1.9*E* − 07	5.4*E* − 07	3.7*E* − 06	**6.3*E ***−** 07**	4.2*E* − 07	5.4*E* − 06	1.2*E* − 06	6.3*E* − 07	3.2*E* − 07
A549-X-ray (1.5 Gy)	Conc.	3.8*E* − 07	3.2*E* − 06	6.9*E* − 06	7.4*E* − 07	6.1*E* − 07	6.2*E* − 06	6.9*E* − 06	3.4*E* − 05	**3.0*E ***−** 05**	5.5*E* − 06	1.1*E* − 04	1.6*E* − 05	7.6*E* − 06	6.3*E* − 06
	SD	4.1*E* − 08	2.8*E* − 06	2.3*E* − 06	1.7*E* − 07	6.4*E* − 08	5.5*E* − 07	6.0*E* − 07	2.9*E* − 06	**1.1*E ***−** 06**	4.1*E* − 07	1.0*E* − 05	9.7*E* − 07	9.4*E* − 07	6.4*E* − 07
	SEM	1.8*E* − 08	1.3*E* − 06	1.0*E* − 06	7.4*E* − 08	2.9*E* − 08	2.5*E* − 07	2.7*E* − 07	1.3*E* − 06	**4.9*E ***−** 07**	1.8*E* − 07	4.6*E* − 06	4.4*E* − 07	4.2*E* − 07	2.9*E* − 07
THP-alpha (0 Gy)	Conc.	3.9*E* − 07	1.1*E* − 05	**1.1*E ***−** 05**	6.6*E* − 07	4.4*E* − 07	1.8*E* − 06	3.7*E* − 06	1.2*E* − 06	1.7*E* − 05	2.7*E* − 06	3.8*E* − 05	7.7*E* − 06	**7.7*E ***−** 06**	**8.9*E ***−** 06**
	SD	7.6*E* − 08	3.2*E* − 06	**2.3*E ***−** 06**	2.7*E* − 07	4.8*E* − 08	5.2*E* − 07	1.4*E* − 06	2.3*E* − 07	6.5*E* − 06	1.1*E* − 06	1.3*E* − 05	1.3*E* − 06	**3.2*E ***−** 06**	**2.4*E ***−** 06**
	SEM	3.4*E* − 08	1.5*E* − 06	**1.0*E ***−** 06**	1.2*E* − 07	2.1*E* − 08	2.3*E* − 07	6.2*E* − 07	1.0*E* − 07	2.9*E* − 06	5.0*E* − 07	5.7*E* − 06	5.8*E* − 07	**1.5*E ***−** 06**	**1.1*E ***−** 06**
THP-alpha (1.5 Gy)	Conc.	4.0*E* − 07	1.5*E* − 05	**7.9*E ***−** 06**	7.4*E* − 07	4.8*E* − 07	1.6*E* − 06	3.9*E* − 06	1.4*E* − 06	1.4*E* − 05	2.8*E* − 06	4.6*E* − 05	9.2*E* − 06	**5.2*E ***−** 06**	**5.9*E ***−** 06**
	SD	4.4*E* − 08	4.9*E* − 06	**1.2*E ***−** 06**	4.0*E* − 07	4.0*E* − 08	3.2*E* − 07	7.6*E* − 07	2.9*E* − 07	1.6*E* − 06	3.0*E* − 07	5.7*E* − 06	9.0*E* − 07	**3.8*E ***−** 07**	**2.7*E ***−** 07**
	SEM	2.0*E* − 08	2.2*E* − 06	**5.6*E ***−** 07**	1.8*E* − 07	1.8*E* − 08	1.4*E* − 07	3.4*E* − 07	1.3*E* − 07	7.1*E* − 07	1.4*E* − 07	2.6*E* − 06	4.0*E* − 07	**1.7*E ***−** 07**	**1.2*E ***−** 07**
THP-X-ray (0 Gy)	Conc.	3.7*E* − 07	**1.1*E ***−** 05**	1.1*E* − 05	1.0*E* − 06	4.4*E* − 07	**1.1*E ***−** 06**	3.8*E* − 06	**9.4*E ***−** 07**	1.5*E* − 05	2.5*E* − 06	3.5*E* − 05	7.7*E* − 06	6.5*E* − 06	8.0*E* − 06
	SD	2.8*E* − 08	**2.6*E ***−** 06**	1.4*E* − 06	1.9*E* − 07	2.4*E* − 08	**3.1*E ***−** 07**	5.2*E* − 07	**1.6*E ***−** 07**	1.8*E* − 06	1.7*E* − 07	3.9*E* − 06	4.4*E* − 07	4.6*E* − 07	7.2*E* − 07
	SEM	1.2*E* − 08	**1.1*E ***−** 06**	6.2*E* − 07	8.6*E* − 08	1.1*E* − 08	**1.4*E ***−** 07**	2.3*E* − 07	**7.3*E ***−** 08**	8.1*E* − 07	7.6*E* − 08	1.8*E* − 06	2.0*E* − 07	2.1*E* − 07	3.2*E* − 07
THP-X-ray (1.5 Gy)	Conc.	3.8*E* − 07	**1.9*E ***−** 05**	9.6*E* − 06	6.0*E* − 07	4.5*E* − 07	**1.8*E ***−** 06**	3.9*E* − 06	**2.1*E ***−** 06**	1.5*E* − 05	2.6*E* − 06	3.4*E* − 05	7.6*E* − 06	5.5*E* − 06	7.4*E* − 06
	SD	1.8*E* − 08	**7.8*E ***−** 06**	1.6*E* − 06	5.0*E* − 07	2.3*E* − 08	**3.6*E ***−** 07**	8.8*E* − 07	**1.7*E ***−** 07**	1.4*E* − 06	3.4*E* − 07	2.0*E* − 06	7.6*E* − 07	6.8*E* − 07	3.0*E* − 07
	SEM	7.8*E* − 09	**3.5*E ***−** 06**	7.0*E* − 07	2.2*E* − 07	1.0*E* − 08	**1.6*E ***−** 07**	3.9*E* − 07	**7.6*E ***−** 08**	6.4*E* − 07	1.5*E* − 07	9.0*E* − 07	3.4*E* − 07	3.0*E* − 07	1.3*E* − 07

**Table 1b:** (b)

Cell type		Cytokines screened												
		Eotaxin	FGF basic	G-CSF	GM-CSF	IFN-g	IP-10	MCP-1	MIP-1a	PDGF-bb	MIP-1b	RANTES	TNF-a	VEGF
A549-alpha (0 Gy)	Conc.	**3.2*E ***−** 05**	4.2*E* − 05	3.0*E* − 06	6.0*E* − 05	**7.9*E ***−** 03**	4.8*E* − 05	**6.0*E ***−** 04**	2.8*E* − 06	**6.8*E ***−** 06**	3.7*E* − 06	6.9*E* − 06	**8.6*E ***−** 06**	**6.1*E ***−** 03**
	SD	**2.9*E ***−** 06**	1.1*E* − 05	1.3*E* − 06	2.1*E* − 05	**2.6*E ***−** 04**	4.7*E* − 06	**3.4*E ***−** 05**	6.4*E* − 07	**9.6*E ***−** 07**	7.1*E* − 07	6.0*E* − 07	**2.4*E ***−** 06**	**4.6*E ***−** 04**
	SEM	**1.3*E ***−** 06**	4.9*E* − 06	5.6*E* − 07	9.6*E* − 06	**1.2*E ***−** 04**	2.1*E* − 06	**1.5*E ***−** 05**	2.8*E* − 07	**4.3*E ***−** 07**	3.2*E* − 07	2.7*E* − 07	**1.1*E ***−** 06**	**2.1*E ***−** 04**
A549-alpha (1.5 Gy)	Conc.	**2.7*E ***−** 05**	4.3*E* − 05	2.7*E* − 06	4.9*E* − 05	**7.3*E ***−** 03**	4.4*E* − 05	**5.1*E ***−** 04**	2.6*E* − 06	**1.3*E ***−** 05**	3.3*E* − 06	6.7*E* − 06	**5.7*E ***−** 06**	**5.0*E ***−** 03**
	SD	**3.5*E ***−** 06**	8.6*E* − 06	1.6*E* − 06	1.9*E* − 05	**3.6*E ***−** 04**	3.4*E* − 06	**2.0*E ***−** 05**	2.2*E* − 07	**3.7*E ***−** 07**	3.6*E* − 07	9.8*E* − 07	**4.3*E ***−** 07**	**1.3*E ***−** 04**
	SEM	**1.6*E ***−** 06**	3.8*E* − 06	7.0*E* − 07	8.7*E* − 06	**1.6*E ***−** 04**	1.5*E* − 06	**8.8*E ***−** 06**	9.7*E* − 08	**1.7*E ***−** 07**	1.6*E* − 07	4.4*E* − 07	**1.9*E ***−** 07**	**6.0*E ***−** 05**
A549-X-ray (0 Gy)	Conc.	**3.3*E ***−** 05**	3.1*E* − 05	2.6*E* − 06	4.9*E* − 05	**7.5*E ***−** 03**	4.4*E* − 05	6.1*E* − 04	2.5*E* − 06	6.4*E* − 06	3.3*E* − 06	6.2*E* − 06	6.4*E* − 06	5.3*E* − 03
	SD	**2.6*E ***−** 06**	4.6*E* − 06	1.2*E* − 06	5.6*E* − 06	**6.7*E ***−** 04**	3.9*E* − 06	3.9*E* − 05	4.0*E* − 07	1.2*E* − 06	5.6*E* − 07	5.2*E* − 07	1.9*E* − 06	4.0*E* − 04
	SEM	**1.2*E ***−** 06**	2.1*E* − 06	5.2*E* − 07	2.5*E* − 06	**3.0*E ***−** 04**	1.8*E* − 06	1.7*E* − 05	1.8*E* − 07	5.2*E* − 07	2.5*E* − 07	2.3*E* − 07	8.3*E* − 07	1.8*E* − 04
A549-X-ray (1.5 Gy)	Conc.	**2.8*E ***−** 05**	2.9*E* − 05	2.5*E* − 06	5.3*E* − 05	**6.2*E ***−** 03**	4.5*E* − 05	6.7*E* − 04	2.4*E* − 06	5.5*E* − 06	3.1*E* − 06	6.3*E* − 06	6.4*E* − 06	4.8*E* − 03
	SD	**3.6*E ***−** 06**	4.0*E* − 06	4.2*E* − 07	4.7*E* − 06	**1.0*E ***−** 03**	4.5*E* − 06	4.2*E* − 05	2.0*E* − 07	8.0*E* − 07	4.3*E* − 07	3.9*E* − 07	1.2*E* − 06	4.5*E* − 04
	SEM	**1.6*E ***−** 06**	1.8*E* − 06	1.9*E* − 07	2.1*E* − 06	**4.5*E ***−** 04**	2.0*E* − 06	1.9*E* − 05	9.0*E* − 08	3.6*E* − 07	1.9*E* − 07	1.7*E* − 07	5.5*E* − 07	2.0*E* − 04
THP-alpha (0 Gy)	Conc.	3.4*E* − 05	2.5*E* − 05	1.6*E* − 04	4.8*E* − 05	6.3*E* − 03	**3.9*E ***−** 05**	6.9*E* − 06	2.7*E* − 06	7.5*E* − 06	**9.2*E ***−** 06**	**1.7*E ***−** 04**	6.6*E* − 06	**8.3*E ***−** 04**
	SD	1.0*E* − 05	9.4*E* − 06	7.9*E* − 05	3.6*E* − 05	2.5*E* − 03	**5.2*E ***−** 06**	3.7*E* − 06	9.9*E* − 07	2.7*E* − 06	**2.4*E ***−** 06**	**2.2*E ***−** 05**	7.5*E* − 06	**2.6*E ***−** 04**
	SEM	4.5*E* − 06	4.2*E* − 06	3.5*E* − 05	1.6*E* − 05	1.1*E* − 03	**2.3*E ***−** 06**	1.7*E* − 06	4.4*E* − 07	1.2*E* − 06	**1.1*E ***−** 06**	**9.7*E ***−** 06**	3.3*E* − 06	**1.1*E ***−** 04**
THP-alpha (1.5 Gy)	Conc.	2.8*E* − 05	2.0*E* − 05	3.2*E* − 04	5.3*E* − 05	6.0*E* − 03	**8.1*E ***−** 05**	4.3*E* − 06	2.3*E* − 06	6.5*E* − 06	**1.5*E ***−** 05**	**2.0*E ***−** 04**	2.7*E* − 06	**1.2*E ***−** 03**
	SD	1.5*E* − 06	2.2*E* − 06	1.6*E* − 04	5.9*E* − 06	7.8*E* − 04	**1.2*E ***−** 05**	2.6*E* − 07	1.1*E* − 07	9.7*E* − 07	**1.5*E ***−** 06**	**1.2*E ***−** 05**	1.1*E* − 06	**2.1*E ***−** 04**
	SEM	6.9*E* − 07	9.8*E* − 07	7.2*E* − 05	2.6*E* − 06	3.5*E* − 04	**5.2*E ***−** 06**	1.2*E* − 07	4.8*E* − 08	4.3*E* − 07	**6.8*E ***−** 07**	**5.3*E ***−** 06**	4.7*E* − 07	**9.5*E ***−** 05**
THP-X-ray (0 Gy)	Conc.	3.3*E* − 05	**2.2*E ***−** 05**	1.8*E* − 04	3.2*E* − 05	6.0*E* − 03	**4.2*E ***−** 05**	4.9*E* − 06	2.3*E* − 06	6.7*E* − 06	1.2*E* − 05	**1.6*E ***−** 04**	3.8*E* − 06	7.5*E* − 04
	SD	3.2*E* − 06	**1.0*E ***−** 06**	6.9*E* − 05	8.7*E* − 06	4.5*E* − 04	**7.6*E ***−** 06**	3.9*E* − 07	1.0*E* − 07	6.1*E* − 07	7.1*E* − 06	**7.5*E ***−** 06**	1.5*E* − 06	5.4*E* − 05
	SEM	1.4*E* − 06	**4.6*E ***−** 07**	3.1*E* − 05	3.9*E* − 06	2.0*E* − 04	**3.4*E ***−** 06**	1.7*E* − 07	4.7*E* − 08	2.7*E* − 07	3.2*E* − 06	**3.3*E ***−** 06**	6.6*E* − 07	2.4*E* − 05
THP-X-ray (1.5 Gy)	Conc.	2.9*E* − 05	**1.9*E ***−** 05**	1.8*E* − 04	3.0*E* − 05	5.2*E* − 03	**8.3*E ***−** 05**	4.9*E* − 06	2.2*E* − 06	6.2*E* − 06	1.4*E* − 05	**2.5*E ***−** 04**	3.3*E* − 06	7.3*E* − 04
	SD	3.0*E* − 06	**1.7*E ***−** 06**	1.9*E* − 04	4.6*E* − 06	1.1*E* − 03	**6.5*E ***−** 06**	4.0*E* − 07	2.3*E* − 07	1.7*E* − 06	9.2*E* − 07	**6.5*E ***−** 06**	2.0*E* − 06	1.3*E* − 04
	SEM	1.3*E* − 06	**7.7*E ***−** 07**	8.3*E* − 05	2.0*E* − 06	5.0*E* − 04	**2.9*E ***−** 06**	1.8*E* − 07	1.0*E* − 07	7.6*E* − 07	4.1*E* − 07	**2.9*E ***−** 06**	8.8*E* − 07	5.7*E* − 05

## Abbreviations

*α*: Alpha
^241^Am: Americium
^222^Rn: Radon
^214^Po: Polonium
FC: Fold change
MD: Mylar-based plastic dishes
FBS: Fetal bovine serum
TBS: Triphosphate buffered saline
TST: TBS serum triton
ELISA: Enzyme-linked immunosorbent assay
PBS: Phosphate buffered saline
EDTA: Ethylenediaminetetraacetic acid
^238^Pu: Plutonium
IL: Interleukin
IFN-*γ*: Interferon-*γ*
GM-CSF: Granulocyte/macrophage colony-stimulating factor
FGF: Fibroblast growth factor
IP-10: Interferon gamma-induced protein 10 kDa
MCP-1: Monocyte chemotactic protein-1
(MIP)-1a: Macrophage inflammatory protein
PDGF: Platelet derived growth factor
RANTES: Regulated upon activation, normal *t*-cell expressed, and secreted
VEGF: Vascular endothelial growth factor
TNF-*α*: Tumour necrosis factor-*α*
WLM: Working level months
RPMI: Royal Park Medical Institute
LET: Linear energy transfer.

## References

[1] Al-Zoughool M, Krewski D (2009). Health effects of radon: a review of the literature. *International Journal of Radiation Biology*.

[2] Caswell RS, Coyne JJ (1990). Microdosimetry of radon and radon daughters. *Radiation Protection Dosimetry*.

[3] Alberg AJ, Ford JG, Samet JM (2007). Epidemiology of lung cancer: ACCP evidence-based clinical practice guidelines (2nd edition). *Chest*.

[4] Goldfarb A, Litvinenko M (2007). *Death of a Dissident: The Poisoning of Alexander Litvinenko and the Return of the KGB*.

[5] U.S. Department of Energy http://www.evs.anl.gov/pub/dsp_detail.cfm?PubID=1472.

[6] Holland JP, Williamson MJ, Lewis JS (2010). Unconventional nuclides for radiopharmaceuticals. *Molecular Imaging*.

[7] Allen BJ (2008). Clinical trials of targeted alpha therapy for cancer. *Reviews on Recent Clinical Trials*.

[8] Jostes RF (1996). Genetic, cytogenetic, and carcinogenic effects of radon: a review. *Mutation Research*.

[9] Wilkins MR, Appel RD, Van Eyk JE (2006). Guidelines for the next 10 years of proteomics. *Proteomics*.

[10] Grivennikov SI, Karin M (2011). Inflammatory cytokines in cancer: tumour necrosis factor and interleukin 6 take the stage. *Annals of the Rheumatic Diseases*.

[11] Narayanan PK, LaRue KEA, Goodwin EH, Lehnert BE (1999). Alpha particles induce the production of interleukin-8 by human cells. *Radiation Research*.

[12] Xu NY, Zhang SP, Nie JH, Li JX, Tong J (2008). Radon-induced proteomic profile of lung tissue in rats. *Journal of Toxicology and Environmental Health*.

[13] Beaton LA, Burn TA, Stocki TJ, Chauhan V, Wilkins RC (2011). Development and characterization of an in vitro alpha radiation exposure system. *Physics in Medicine and Biology*.

[14] Arya M, Patel HRH (2003). Expanding role of chemokines and their receptors in cancer. *Expert Review of Anticancer Therapy*.

[15] Braeuer RR, Zigler M, Villares GJ, Dobroff AS, Bar-Eli M (2011). Transcriptional control of melanoma metastasis: the importance of the tumor microenvironment. *Seminars in Cancer Biology*.

[16] Appelmann I, Liersch R, Kessler T, Mesters RM, Berdel WE (2010). Angiogenesis inhibition in cancer therapy: platelet-derived growth factor (PDGF) and vascular endothelial growth factor (VEGF) and their receptors: biological functions and role in malignancy. *Recent Results in Cancer Research*.

[17] Feghali CA, Wright TM (1997). Cytokines in acute and chronic inflammation. *Frontiers in Bioscience*.

[18] Goswami R, Kaplan MH (2011). A brief history of IL-9. *Journal of Immunology*.

[19] Gleich GJ (2000). Mechanisms of eosinophil-associated inflammation. *Journal of Allergy and Clinical Immunology*.

[20] Schroder K, Hertzog PJ, Ravasi T, Hume DA (2004). Interferon-*γ*: an overview of signals, mechanisms and functions. *Journal of Leukocyte Biology*.

[21] Pinto M, Prise KM, Michael BD (2002). Double strand break rejoining after irradiation of human fibroblasts with X rays or *α* particles: PFGE studies and numerical models. *Radiation Protection Dosimetry*.

[22] Chauhan V, Howland M, Chen J, Kutzner B, Wilkins RC (2011). Differential effects of alpha-particle radiation and X-irradiation on genes associated with apoptosis. *Radiology Research and Practice*.

[23] Tugues S, Koch S, Gualandi L, Li X, Claesson-Welsh L (2011). Vascular endothelial growth factors and receptors: anti-angiogenic therapy in the treatment of cancer. *Molecular Aspects of Medicine*.

